# Spatiotemporal heterogeneity of neutrophil extracellular traps in hepatocellular carcinoma microenvironment and targeted therapy progress

**DOI:** 10.1186/s12967-026-08381-x

**Published:** 2026-06-08

**Authors:** Ying Zheng, Ganggang Jia, Bin Li, Chuyi Li, Jiucong Zhang, Xiaohui Yu

**Affiliations:** 1 College of Integrated Traditional Chinese and Western Medicine, Gansu University of Chinese Medicine, LanZhou, GanSu China; 2 The 940th Hospital of the Joint Logistics Support Force of the Chinese People’s Liberation Army, LanZhou, GanSu China

**Keywords:** Neutrophil extracellular traps, Hepatocellular carcinoma, Spatiotemporal heterogeneity, Targeted therapy

## Abstract

**Background:**

Neutrophil extracellular traps (NETs) form a spatiotemporally heterogeneous defensive architecture within the tumor microenvironment (TME), representing a newly identified multi-dimensional biological barrier system. Particularly in hepatocellular carcinoma (HCC), this system drives the heterogeneous evolution of the TME through the integration of mechanical stress transduction (YAP/TAZ activation) and protease-mediated signaling network remodeling (reconfiguration of intercellular signaling via proteolytic enzymes like MMP9, e.g. the MMP9/TGF-β axis).

**Main body:**

The NET-driven heterogeneous evolution of the HCC TME is characterized by region-specific epithelial-mesenchymal transition (EMT) progression, metabolic reprogramming of tumor cells, and the formation of immunosuppressive niches within the TME. These changes collectively reshape the TME’s biological properties, alter the energy landscape of tumor cells (a framework describing dynamic metabolic/functional states of tumor cells shaped by microenvironmental cues), and maintain their stem cell properties, ultimately contributing to therapeutic heterogeneity and cross-regional drug resistance. Existing targeted therapeutic strategies against NETs in HCC and their clinical translational potential are analyzed herein.

**Conclusions:**

This review systematically synthesizes the spatiotemporal heterogeneity of NETs in the HCC microenvironment and their functional roles, providing new insights into HCC treatment.

## Introduction

Primary liver cancer ranks third among the leading causes of cancer-related mortality worldwide, with an escalating annual incidence that exacerbates the global healthcare burden [1, 2]. Hepatocellular carcinoma (HCC), the predominant histological subtype, accounts for 75–85% of primary liver cancer cases [1]. Its pathogenesis is intricately linked to multiple risk factors: chronic infection by hepatitis B virus (HBV) or hepatitis C virus (HCV) contributes to approximately 80% of global HCC cases [3]; alcohol consumption exceeding 70 grams per day for over 10 years increases the risk [4]; non-alcoholic liver disease (NAFLD) has emerged as a rising cause, particularly in regions with high obesity prevalence [4]; and exposure to aflatoxin B1, a potent carcinogen in mold-contaminated food, is strongly associated with HCC development in sub-Saharan Africa and Southeast Asia [5].

Despite significant advances in therapeutic modalities, including curative-intent surgical resection [6], liver transplantation [7], transarterial chemoembolization (TACE) [8], radiofrequency ablation (RFA) [9], and systemic therapies such as tyrosine kinase inhibitors (TKIs) and immune checkpoint inhibitors (ICIs) [10, 11], the clinical management of HCC remains challenging. The incidence of HCC tumor recurrence is as high as 88% even among patients who have undergone curative-intent treatment, primarily due to microscopic residual disease and intrahepatic metastasis [12]. Systemic immunotherapy, despite its revolutionary impact on oncology, faces significant limitations in HCC clinical management. The objective response rate to immune checkpoint inhibitor (ICI) therapy remains below 20%, while immune-related adverse events (irAEs) triggered by ICI-mediated immune activation pose a critical clinical challenge [13].

The tumor microenvironment (TME) of HCC is characterized by profound immunosuppression, driven by the coordinated actions of diverse immune cell populations. A high infiltration of myeloid-derived suppressor cells (MDSCs), tumor-associated macrophages (TAMs), and regulatory T cells (Tregs) creates a cytokine-rich milieu that actively suppresses cytotoxic T lymphocyte (CTL) function [14, 15]. Notably, recent studies indicate that neutrophils, traditionally considered short-lived effector cells, exhibit plastic phenotypes within the HCC TME [16–18]. They can either promote tumor progression by secreting pro-angiogenic factors and matrix metalloproteinases or participate in anti-tumor responses through direct tumor cell killing or immune activation [19, 20]. This dualistic role underscores the need for a comprehensive understanding of neutrophil biology and its interactions with other immune components, which has emerged as a critical frontier in HCC immunotherapy research [21].

## Neutrophil extracellular traps (NETs)

As the most abundant leukocytes in humans, neutrophils employ a multi-faceted arsenal to combat pathogens, integrating phagocytosis [22], reactive oxygen species (ROS) generation [23], granule enzyme release [24], immune cell recruitment [25], and the formation of NETs [26]. NETs represent a specialized defense strategy, manifesting as web-like extracellular structures composed of decondensed DNA backbones decorated with histones (e.g., citrullinated H3/H4) [27, 28], granular proteases (neutrophil elastase, myeloperoxidase) [29, 30], and antimicrobial peptides (LL-37, lactoferrin) [31]. Triggered by oversized or overwhelming pathogens, NET formation (NETosis) involves a tightly regulated cascade [32]: chromatin remodeling via ROS-mediated activation of peptidylarginine deiminase 4 (PAD4) to drive histone citrullination and decondense nuclear DNA [33], cytoskeletal disruption through neutrophil elastase-mediated degradation of actin filaments to impair motility and phagocytosis [32], and membrane permeabilization via gasdermin-D polymerization to facilitate plasma membrane rupture and release NETs alongside granule cytotoxic cargo [32]. This process typically results in neutrophil lytic death, though non-lytic NET release pathways have been documented [26].

Accumulating evidence strongly supports the direct and indirect regulatory effects of NETs on both adaptive and innate immunity, underscoring their pivotal role in maintaining immune homeostasis [34]. NETs are dynamically formed and exhibit antibacterial activity in various infectious conditions, including bacterial, parasitic, and fungal infections, where pathogens serve as stimuli to induce NET formation [35, 36]. NET-induced damage has been implicated in multiple disorders, including systemic lupus erythematosus [37], periodontitis [38], atherosclerosis [39], thrombosis [40], acute lung injury [41], and sepsis [42]. However, like a double-edged sword, persistent inflammation or continuous stimulation leads to excessive NET formation, exacerbating tissue damage during dysregulated inflammatory responses [43]. Additionally, dysregulated NET formation is observed in non-pathogenic conditions, including but not limited to sterile inflammation, autoimmune diseases, metabolic disorders, vasculitis, thrombogenesis, and carcinogenesis [43, 44]. Current evidence suggests that NETs play dual roles in these non-pathogenic conditions. On one hand, in autoimmune diseases, NETs act as autoantigens, contributing to tissue destruction, amplifying inflammatory cascades, and promoting thrombosis [44–46]. On the other hand, aggregated NETs formed during sterile inflammation, containing diverse enzymes, function as inflammatory mediators by degrading proinflammatory cytokines and chemokines, thereby promoting inflammation resolution and wound healing [47, 48].

### Pro-tumorigenic roles of NETs in the tumor microenvironment

NETs have emerged as critical modulators of cancer progression, with accumulating evidence highlighting their multifaceted pro-tumorigenic functions [43]. A pan-cancer analysis of the Nod-like receptor signaling pathway demonstrated that tumor-promoting immune infiltration (e.g., elevated immature B cells in high-risk groups) is closely associated with aggressive tumor behavior, a phenomenon consistent with NETs-induced immunosuppressive niche formation in HCC [49]. This suggests that NETs may regulate intra-tumoral immune cell distribution through similar signaling cascades, thereby facilitating EMT and metastasis. The DNA-histone complexes of NETs, decorated with proteases like neutrophil elastase (NE) and myeloperoxidase (MPO) [50, 51], drive tumor development through three interconnected mechanisms: microenvironment remodeling, immune evasion, and metastatic promotion [26, 43, 52].

In pancreatic ductal adenocarcinoma (PDAC), collagen binding to discoid domain receptor 1 (DDR1) on tumor cells induces CXCL5 secretion, recruiting neutrophils to form NETs that enhance cancer cell invasion via matrix degradation [53]. Similarly, in breast cancer, tumor-derived cathepsin C promotes metastasis through NET-dependent degradation of thrombospondin-1 (TSP-1), a potent metastasis suppressor [54, 55]. These findings illustrate how NET-associated proteases directly facilitate tumor cell motility.

The premetastatic niche formation represents another pivotal role of NETs. In orthotopic breast cancer models, aged neutrophils release NETs containing mitochondrial DNA, remodeling lung tissue to favor metastatic colonization [56]. Mechanistically, lung-resident mesenchymal stromal cells (LMSCs) in MMTV-PyMT mice promote NET formation via a complement 3 (C3)-STAT6 axis, creating a permissive microenvironment for cancer cell seeding [55]. Notably, oxidized albumin-induced lung-specific NET deposition has been linked to the organotropism of head and neck/breast cancer metastases, underscoring the spatial regulation of NET-mediated metastasis [57].

### NETs as architects of immunosuppressive tumor niches

NETs profoundly reshape the immune landscape of tumors, establishing a microenvironment that stifles anti-cancer immunity [58–60]. The physical meshwork of NET-DNA acts as a barrier, impeding cytotoxic T cell and natural killer cell access to cancer cells [26, 43]. This effect is compounded by PD-L1 embedded within NETs, which induces T cell exhaustion in liver metastasis models [61]. Additionally, NETs drive regulatory T cell (Treg) differentiation through TLR4-mediated metabolic reprogramming, as seen in NAFLD-related hepatocellular carcinoma [62–64]. Clinical evidence supports these mechanisms: bladder cancer patients with radiation resistance exhibit high intratumoral neutrophil-to-CD8+ T cell ratios, a phenotype reversed by DNase I digestion of NETs [43, 65, 66]. In triple-negative breast cancer, metastatic lesions contain elevated NETs, coinciding with reduced T cell infiltration [60]. These observations highlight NETs as critical mediators of immune evasion, linking neutrophil heterogeneity to therapeutic resistance [60, 67–69].

### Clinical correlates and Prognostic value of NETs

Circulating and tissue NET markers show strong associations with cancer progression and poor outcomes. In HCC, plasma levels of MPO-DNA complexes and citrullinated histone 3 (CitH3) in primary tumors correlate with extrahepatic metastasis and shortened survival [70, 71]. Breast cancer patients with metastatic disease exhibit higher serum NE-DNA complexes than those with localized tumors, while esophageal-gastric cancer patients with lymph node involvement have elevated NET levels [72].

Immunostaining studies further validate NETs’ clinical relevance: ovarian cancer omentum from high-stage patients shows increased NET formation (H3Cit+MPO+), while lung metastatic lesions from breast cancer patients display NET deposition preferentially in triple-negative subtypes [55, 73, 74]. These findings establish NETs as potential prognostic biomarkers, though standardization of detection methods (e.g., CitH3 ELISA vs. NET immunofluorescence) remains a key challenge for clinical translation [75–77].

Circulating NET-related biomarkers, including MPO-DNA complexes and CitH3, together with molecular imaging modalities such as ^68^Ga-labeled anti-CitH3 positron emission tomography-magnetic resonance imaging (PET-MRI), exhibit substantial clinical potential for the management of HCC. Specifically, in high-risk populations (e.g., patients with HBV-related cirrhosis), a plasma CitH3 level exceeding 2.1 ng/mL has been shown to predict HCC progression with a moderate discriminative ability. To minimize inter-laboratory variability and ensure the reliability of these diagnostic tools, standardization of pre-analytical procedures and analytical protocols (e.g., utilization of calibrated ELISA kits for biomarker quantification) is imperative. Moreover, a two-step diagnostic algorithm integrating initial screening with NET-related biomarkers followed by confirmatory ^68^Ga-labeled anti-CitH3 PET-MRI for biomarker-positive cases has been proposed to enhance the early detection of small HCC lesions (<2 cm), which are often missed by conventional imaging modalities. Despite these promising advancements, current NET-based diagnostic strategies still face limitations, including potential interference from concurrent infections and insufficient specificity for HCC. Therefore, future research efforts should focus on developing combined diagnostic panels integrating NET-related biomarkers with circulating tumor DNA (ctDNA) and AFP-L3 to improve diagnostic specificity, as well as advancing point-of-care testing technologies to facilitate the application of NET-based diagnostics in resource-limited settings.

### Targeting NETs: emerging therapeutic strategies

The pro-tumorigenic functions of NETs have spurred the development of targeted therapeutic approaches [78]. DNase I, which degrades NET-DNA, reduces metastasis in preclinical breast and gastric cancer models [79, 80]. A phase II trial evaluating recombinant DNase 1 in advanced solid tumors showed promising safety and survival benefits, particularly in patients with high baseline NET markers [81]. Inhibitors of NET formation represent another avenue: PAD4 antagonist Cl-amidine decreases NET release in HCC models [82], synergizing with PD-1 blockade to enhance T cell responses in colorectal cancer [83]. NE inhibitors (e.g., sivelestat) suppress NET-mediated angiogenesis in PDAC [53, 84], while RAGE receptor antagonists block NET-induced pancreatic stellate cell activation [85, 86]. Clinical trials combining NET-modifying agents with chemotherapy or immunotherapy (e.g., NCT04602007) are underway, aiming to overcome treatment resistance [87, 88]. A brief overview of representative NET-targeted agents is summarized in Table 1.

**Table 1:** Summary of NET-targeted therapeutic agents in preclinical and clinical development

Category	Agent	Stage	Disease model/indication	Key mechanism related to NETs
NET degradation	DNase I	Phase II	Advanced solid tumors	Degrades NET-DNA backbone
NET formation inhibitor	PAD4 inhibitor (Cl-amidine)	Preclinical	HCC, colorectal cancer	Blocks histone citrullination and NET formation
Neutrophil elastase inhibitor	Sivelestat	Preclinical	Pancreatic cancer	Inhibits NE-mediated angiogenesis and NET activity
Signaling antagonist	RAGE antagonist	Preclinical	Pancreatic stellate cell model	Blocks NET-induced pro-tumor signaling

Over the past decade, NETs, initially recognized as an effector mechanism centered on pathogen clearance within the innate immune system [89], have gradually been unveiled as multi-dimensional pathophysiological regulatory nodes in the field of tumor biology [90, 91]. However, a systematic elucidation of their spatiotemporal heterogeneous roles in HCC remains lacking. Existing studies have mostly focused on the pro-inflammatory and pro-fibrotic functions of NETs [64, 89]. However, the molecular mechanisms by which NETs drive TME heterogeneity remain poorly understood. Specifically, it is unclear how NETs integrate (1) mechanosignal transduction via YAP/TAZ-Hippo pathway [92], (2) matrix-remodeling protease network [93] such as MMP9/TGF-β axis-mediated ECM degradation pathway [94] and (3) the TGF-βR/SMAD signal cascade [95]. These coupled biomechanical and biochemical signals ultimately build a multi-dimensional (molecule-cell-tissue) barrier that drives the heterogeneous evolution of the TME. As downstream effectors of the Hippo pathway, the YAP/TAZ signaling axis exerts its regulatory functions primarily through the integration of mechanical cues and NET-mediated modulation. Across distinct stages and regional compartments of HCC, YAP/TAZ senses extracellular matrix (ECM) stiffness dynamics via the integrin αvβ3-FAK-YAP/TAZ mechanotransduction axis, enabling context-dependent adaptation of tumor cell behaviors [96, 97]. In contrast, the MMP9/TGF-β signaling axis is defined by hallmark bidirectional crosstalk and epigenetic regulation, which collectively underpin its pivotal role in driving HCC invasion and metastasis [98]. Notably, these two pathways exhibit prominent spatial synergy: YAP/TAZ dominates the tumor core, where it orchestrates therapeutic resistance and maintains cancer stem cell (CSC) stemness [99]; meanwhile, the MMP9/TGF-β axis governs the invasive margin to promote local tumor dissemination [100]. This spatiotemporally segmented collaboration not only shapes the intratumoral heterogeneity of HCC but also provides critical signaling support for the multistep progression of this malignancy (Table 2). Notably, this spatiotemporal heterogeneity not only manifests as differences in the physical distribution of NETs but also endows tumor cells with multi-dimensional escape capabilities by dynamically regulating the EMT gradient [101], metabolic plasticity, and the regionalized characteristics of immunosuppressive niches [102, 103]. For instance, NETs enriched around the portal vein can integrate mechanical stress and protease activity to remodel the local ECM rigidity [104, 105] and lock immune checkpoints; while NETs at the edge of cancer nests regulate glutaminolysis through metabolic reprogramming to maintain the stemness reservoir of cancer stem cells (CSCs) [106]. These findings suggest that the “niche topological network” constructed by NETs may be a potential root cause of the treatment heterogeneity of HCC.

**Table 2:** Core information on the YAP/TAZ and MMP9/TGF-β pathway

Signaling Pathway	Core Mechanisms	Interaction with NETs	Clinical Implications in HCC
YAP/TAZ	1) Downstream of Hippo pathway and nuclear translocation upon dephosphorylation 2) Forms complex with TEAD transcription factors 3) Induces SLC7A11 expression (cooperates with ATF4) to repress ferroptosis 4) Activates oncogenes (CTGF, CYR61) promoting proliferation and invasion	NETs-derived IL-8 inhibits Hippo pathway kinases, enhancing YAP/TAZ activation and forming a regulatory loop	1) Mediates sorafenib resistance 2) Potential target for reversing therapy resistance 3) High expression correlates with poor prognosis
MMP9/TGF-β	1) MMP9 cleaves latent TGF-β to trigger activation; TGF-β synergizes with TNF-α to upregulate MMP9 2) Signaling mediated by TGF-βRI/Smad3 and p38 pathways 3) Epigenetic regulation by H3K36me2 at MMP9 promoter 4) MMP9 degrades ECM; TGF-β induces EMT and angiogenesis	NETs-derived NE upregulates MMP9 expression and accelerates TGF-β activation	1) Drives invasion and intrahepatic metastasis 2) H3K36me2 inhibitor may block pathway activation 3) Co-expression indicates high metastatic risk

**Fig. 1:**
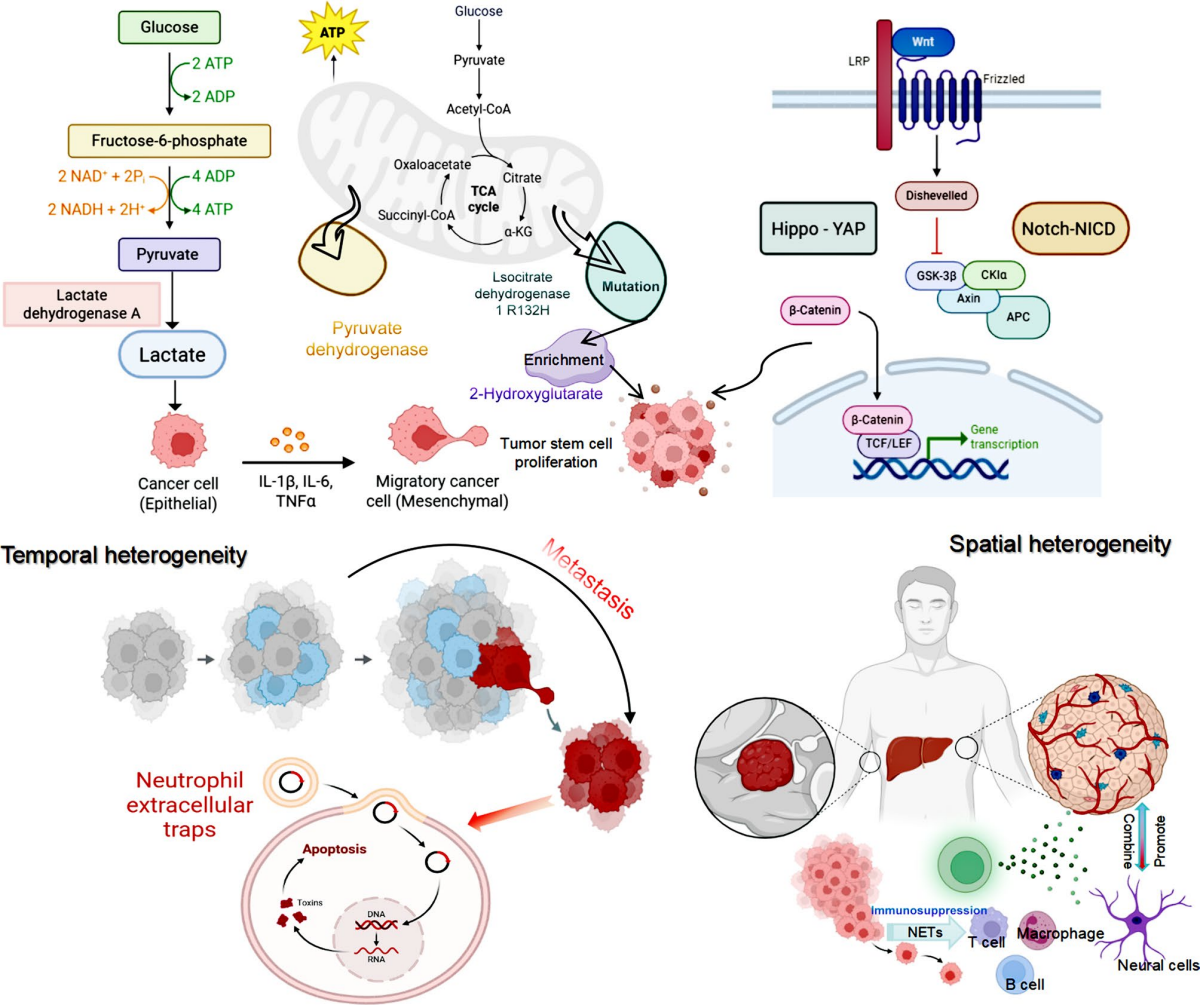
Spatiotemporal heterogeneity of neutrophil extracellular traps in hepatocellular carcinoma. In energy metabolism pathways: glucose is converted to pyruvate by hexokinase (HK); under hypoxia or glycolysis dominance, lactate dehydrogenase a (LDHA) catalyzes pyruvate to lactate, inducing tumor microenvironment (TME) acidification that promotes epithelial-mesenchymal transition (EMT) and tumor invasiveness/metastasis; pyruvate enters mitochondria to participate in the tricarboxylic acid (TCA) cycle and electron transport chain (ETC) via pyruvate dehydrogenase (PDH) and isocitrate dehydrogenase (IDH) for ATP production, while IDH mutations (e.g., IDH1 R132H in liver cancer) cause 2-hydroxyglutarate (2HG) accumulation, triggering epigenetic reprogramming to maintain cancer stem cell (CSC) stemness. In stemness maintenance signaling pathways: normal Hippo pathway activity suppresses YAP/TAZ nuclear translocation, whereas pathway inactivation in tumors activates YAP/TAZ, which binds transcription factors to drive proliferation-, survival-, and stemness-related gene expression; in normal cells, β-catenin is degraded by a complex (GSK-3β, axin, CK1α, APC), while wnt activation in tumors inhibits this degradation, enabling β-catenin nuclear translocation and TCF/LEF-mediated target gene activation to sustain CSC properties; notch receptor-ligand binding induces proteolytic cleavage to release the notch intracellular domain (NICD), which translocates to the nucleus and regulates downstream genes to control CSC stemness and tumor proliferation. For spatiotemporal heterogeneity: temporal heterogeneity manifests as stage-specific changes-precancerous lesions show initial genetic/epigenetic alterations and immune escape; carcinoma in situ forms localized lesions with extracellular matrix (ECM) remodeling; local advanced tumors invade adjacent tissues with increased angiogenesis and recruitment of tumor-associated macrophages (TAMs)/cancer-associated fibroblasts (CAFs); distant metastasis involves tumor cell dissemination, adaptation to new microenvironments, and new focus establishment. Spatial heterogeneity includes distinct tumor regions-the core has insufficient oxygen/nutrients, slow proliferation, dense ECM, and limited immune infiltration; the periphery has adequate nutrients, rapid proliferation, increased invasiveness, and immunosuppressive factor secretion; immune-infiltrated regions contain T/B cells and macrophages (inhibited by tumor cells for immune escape); neural-infiltrated regions show tumor-neural cell interactions promoting growth/metastasis and impairing neural function. Additionally, CSCs and mesenchymal migratory tumor cells secrete IL-1β, IL-6, and TNFα to remodel the TME, while neutrophil extracellular traps (NETs) enhance immunosuppression and apoptosis resistance, collectively driving tumor progression and therapeutic resistance

From the perspective of spatiotemporal biology, this paper systematically analyzes the dynamic distribution patterns of NETs in the HCC microenvironment, their multimodal regulatory mechanisms, and their multimodal interaction networks with TME components (such as cancer-associated fibroblasts (CAFs), tumor-associated macrophages (TAMs), tumor-associated neutrophils (TANs), etc.). It further focuses on the current intervention strategies targeting NETs: including DNase-mediated NETs clearance [107], PAD4 inhibitor-blocked NETs generation [108], and precise intervention at mechanosignal transduction targets (YAP/TAZ-TEAD) [109]. Based on the translational bottlenecks of preclinical models and the application potential of emerging biomaterials (such as spatiotemporal-responsive nanodrug delivery systems [110], a multi-dimensional synergistic treatment paradigm is proposed. This paper aims to construct a multi-dimensional regulatory network of the spatiotemporal dynamic evolution of NETs during HCC progression by integrating single-cell spatial transcriptomics, organoid co-culture models, and computational mechanics simulation techniques, providing theoretical and technical pivots for breaking through the existing treatment predicaments (Fig. 1).

## The dynamic regulatory pattern of time heterogeneity of the NETs in the HCC microenvironment and its molecular targeted intervention

### The dynamic changes and regulatory patterns of NETs in different stages of HCC

#### Precancerous lesion stage (chronic hepatitis/cirrhosis stage)

Chronic hepatitis can be categorized by etiology into chronic hepatitis B, chronic hepatitis C, autoimmune hepatitis, alcoholic liver disease, and drug-induced liver injury. Approximately 250 million people worldwide suffer from chronic hepatitis B virus infection [111]. Chronic hepatitis B confers an increased risk of liver cirrhosis and HCC, with nearly one million annual deaths attributable to these sequelae [111, 112]. During the progression from chronic hepatitis and liver cirrhosis to HCC, NET formation is driven by the chronic inflammatory microenvironment (proinflammatory cytokines such as IL-8 and TNF-α) and reactive oxygen species (ROS) released by damage-associated molecular patterns (DAMPs) from injured hepatocytes, triggering NETosis via activation of the NADPH oxidase (NOX2)-PAD4 pathway within neutrophils [113]. NETs promote fibrotic matrix remodeling through MMP9/TIMP1 imbalance-induced aberrant activation of hepatic stellate cells (HSCs) and collagen deposition [64]. Concurrently, released high-mobility group box 1 protein (HMGB1) binds to Toll-like receptor 9 (TLR9) on macrophages, driving polarization toward M2-type tumor-associated macrophages (M2-TAMs) and establishing an immunosuppressive niche characterized by PD-L1 upregulation and CD8+ T cell exhaustion [114]. Prolonged inflammatory stimulation further induces epigenetic reprogramming of neutrophils (e.g., DNMT1-mediated DNA hypermethylation) [115], lowering the NETosis threshold to enhance sensitivity to low-intensity stimuli and forming an “inflammation-NETs-fibrosis/immunosuppression” positive feedback loop that ultimately facilitates HCC development.

#### In situ carcinoma (early-stage HCC)

The progression from chronic liver injury to early-stage HCC is characterized by a critical window of opportunity for curative intervention [116]. Early-stage HCC usually has no specific symptoms and can only be detected through imaging examinations or biomarker screenings, which poses a significant challenge for timely diagnosis [117]. Mechanistically, early-stage HCC arises from a series of epigenetic and genetic alterations in hepatocytes within a pre-malignant microenvironment [118, 119]. At the carcinoma in situ stage, the generation of NETs is regulated by both the recruitment of neutrophils mediated by tumor-derived chemokines (G-CSF, CXCL5) [120] and the HIF-1α/PAD4 axis driven by the hypoxic microenvironment [121]. The latter promotes NETosis by inducing citrullination of histone H3 (CitH3) [122]. Consistent with the strategy of mapping molecular signatures to microenvironmental gradients in pan-cancer studies [123], spatial analysis of HCC tissues has confirmed that YAP/TAZ activation is positively correlated with ECM stiffness and NET deposition in the tumor core region, which further promotes the expression of stemness markers (e.g., EpCAM) in tumor cells [123]. At the same time, the released succinate inhibits the mitochondrial complex II activity in CD8+ T cells through the SUCNR1–GPR91 signaling axis [124], leading to ATP depletion and exhaustion phenotype (PD-1+/TIM-3+). As the tumor progresses, NETs exhibit temporal and spatial evolution at the tumor margin: in the early stage, NETs form a physical isolation barrier through the cross-linking of NE and fibrinogen [125], while in the late stage, NETs disrupt the integrity of the basement membrane through the degradation of laminin mediated by tissue proteinase G [126], in synergy with the MMP9/TGF-β1 axis, promoting local invasive growth [127], ultimately forming a malignant cycle that promotes metastasis.

#### Progression stage/metastasis stage (advanced HCC)

During the advanced stage of HCC with extensive metastasis, NET formation is dually driven by therapy-induced DNA damage (e.g., chemotherapy/radiation activating the cGAS-STING pathway [128, 129] and metastatic niche metabolic stress (lactate via the GPR81-p38 MAPK axis [130], creating a cascading amplification effect. NETs recruit myeloid-derived suppressor cells (MDSCs) through the CXCL12–CXCR4 chemokine axis [131, 132], establishing a pre-metastatic niche characterized by VEGF-A upregulation and enhanced vascular permeability [133, 134]. Concurrently, their encapsulated DNA-LL37 antimicrobial peptide complexes activate the TLR9–PI3K/Akt-mTORC1 signaling axis in tumor cells [135], inducing EMT and clonal expansion of CSCs (CD133+/EpCAM+). With sustained therapeutic pressure, NETs undergo functional phenotypic switching: early pro-inflammatory NETs (high in IL-1β/IL-6) are gradually replaced by immunosuppressive NETs (enriched in ARG1/PD-L1) [136], which remodel the CXCR4–SDF1α chemotactic gradient [137] and collagen topological orientation [138] in distal organ microenvironments (e.g., lung, bone) via exosomal delivery of non-coding RNAs such as miR-21/155, ultimately forming a cross-regional vicious cycle and therapeutic resistance. Notably, this dynamic interplay between NETs and the metastatic microenvironment introduces therapeutic vulnerabilities. Emerging evidence suggests that targeting NETosis via PAD4 inhibitors or DNase I could disrupt the CXCL12–CXCR4 axis and normalize vascular permeability, potentially abrogating pre-metastatic niche formation [139, 140]. Additionally, blocking TLR9 or mTORC1 signaling might counteract NET-induced EMT and cancer stem cell expansion [82, 141, 142]. The exosomal miR-21/155-mediated remodeling of distal niches further implicates NETs as systemic regulators, highlighting the need for combinatorial strategies that integrate NET-targeted agents with standard therapies to break the cross-organ metastatic cycle in advanced HCC.

### Molecular targeted intervention strategies based on temporal heterogeneity

#### Temporal-specific NETs clearance strategy

Depending on disease progression, time-specific strategies for NET clearance are as follows: During the early tumor stage, targeting peptidylarginine deiminase 4 (PAD4), a key upstream enzyme in NET formation responsible for histone citrullination via selective inhibitors such as BB-Cl-amidine (to block its catalytic activity) [143], or locally delivering DNase I-functionalized nanoparticles (e.g., pegylated liposome-encapsulated rhDNase I) [144], can directly degrade the DNA scaffold of NETs, thereby inhibiting the formation of NET-mediated profibrotic feedforward loops and proinflammatory microenvironments. In the advanced/metastatic phase, combinatorial intervention is required: NE inhibitor Sivelestat suppresses sustained NET release [145], while concurrent administration of PD-1/PD-L1 immune checkpoint blockers (e.g., Pembrolizumab) [146] reverses NET-induced CD8^+^ T-cell exhaustion (characterized by the PD-1+/TIM-3+/TOX+ phenotype) via the HMGB1–TLR9 axis [147, 148] and reshapes antitumor immune responses. This dual-pathway synergy significantly reduces infiltration of immunosuppressive cells (MDSCs, Tregs) within metastases and enhances immunotherapeutic efficacy by restoring T-cell mitochondrial oxidative phosphorylation. The formulation of time-specific strategies necessitates integration of single-cell dynamic tracing technologies [149, 150] and artificial intelligence-driven dose optimization models [151, 152] to maximize the therapeutic window, ultimately paving the way for precision NET-targeted interventions tailored to the spatiotemporal dynamics of tumor-immune crosstalk.

#### Dynamic signal network Intervention

Cells sense and respond to environmental cues via intricate signaling networks, which undergo spatiotemporal remodeling under stimuli [153, 154]. Dynamic Signal Network Intervention targets key nodes/pathways (via pharmacology, gene editing, etc.) to treat signaling-dysregulated diseases (e.g., cancer) or reverse pathological behaviors [153, 155]. For mechanical signal intervention, small-molecule inhibitors such as the Hippo pathway antagonist Verteporfin [155] can block YAP/TAZ nuclear translocation, while integrin αvβ3 antagonists like Cilengitide [156] interfere with NETs-ECM focal adhesion formation [157], thereby disrupting mechanical signal transduction. Concurrently, matrix stiffness-responsive nanodelivery systems (e.g., MMP2/9-sensitive micelles) [158] have been developed to enable mechanical microenvironment-dependent drug activation by selectively releasing dual PAD4/NE inhibitors in regions of high ECM rigidity [159]. Metabolically, targeting the succinate-SUCNR1 axis mitigates immunosuppression: tumor-derived succinate (enriched in hypoxia) binds CD8+ T cell SUCNR1, hyperactivating mitochondrial SDH and inducing ROS dysfunction [160], while SUCNR1 antagonist NF-56-EJ40 [161, 162] restores CD8+ T cell effector function [163]. Additionally, metformin inhibits LDHA to reduce lactate production/secretion by tumor cells/neutrophils [164, 165], and GPR81 antagonist NPS-2143 blocks lactate-GPR81-p38 MAPK signaling [166], breaking the lactate-NETosis-acidification feedback loop.

This multimodal strategy synergistically decouples mechanical/metabolic signals, reverses NET-driven fibrosis-immunosuppression cycles, enhances intervention specificity, and enables context-aware therapy adapting to TME heterogeneity.

#### Precise timing treatment technology

Temporal precision therapeutics (TPT) represents a paradigm shift in medicine, leveraging real-time molecular dynamics and spatiotemporal biomarker fluctuations to optimize treatment timing, dosing, and combination strategies. Unlike static therapeutic approaches, TPT integrates multi-omics data (genomics, transcriptomics, proteomics, and metabolomics) with dynamic imaging and biosensor technologies to capture the evolving nature of diseases, such as cancer, neurodegenerative disorders, and autoimmune conditions [167].

TPT for NET-associated pathologies necessitates the integration of real-time monitoring and intelligent drug delivery systems. Liquid biopsy techniques enable the quantification of circulating myeloperoxidase (MPO)-DNA complexes and citrullinated histone H3 (CitH3), while NET-specific molecular imaging probes (e.g., ^6 8^ Ga-labeled anti-CitH3 monoclonal antibody PET-MRI) [168, 169] resolve the spatiotemporal heterogeneity of NET formation. Building upon this, microenvironment-responsive nanotherapeutics have been engineered: pH/ROS dual-sensitive polymer carriers (such as poly(β-amino ester)-PEG) [170, 171] trigger drug release in NET-enriched regions, co-delivering DNase I for local NET scaffold degradation [171] and sivelestat for neutrophil elastase inhibition [145]. Furthermore, integrating chronotherapeutic release technologies enables activation of drug-loaded systems during NET “flare-up” periods induced by radiotherapy or chemotherapy (48–72 hours post-treatment), enhancing the delivery efficiency of NET inhibitors like BB-Cl-amidine [172]. This closed-loop “monitoring-imaging-intervention” system transcends the spatiotemporal limitations of conventional therapies, offering a modular framework adaptable to diverse disease contexts.

## The multi-dimensional regulatory mechanisms and specific regulatory strategies of the spatial heterogeneity of NETs in the HCC microenvironment

### Spatial heterogeneity of NETs in the HCC microenvironment

#### Tumor core region

Within the core region of HCC, NETs exhibit spatially heterogeneous enrichment, predominantly localizing to the periphery of hypoxic necrotic zones. Here, they collaborate with M2-polarized tumor-associated macrophages (TAMs), α-smooth muscle actin (α-SMA), and CAFs to form a dense fibrotic barrier [173]. NETs drive this pathogenic niche through multifaceted mechanisms: released CitH3 activates the TLR4/NF-κB pathway in macrophages [174], inducing upregulated PD-L1 expression [175]; concurrently, their associated NE degrades tyrosine kinase inhibitors (TKIs) such as anlotinib [176], fostering drug resistance. Collectively, these interactions establish a “fibrosis-immunosuppression-stem cell sanctuary” tripartite malignant microenvironment. This interconnected network underscores the need for spatially targeted interventions that co-disrupt NET-mediated fibrotic remodeling, immune checkpoint induction, and stem cell niche preservation. Emerging strategies, such as dual TLR4/NE inhibitors conjugated to hypoxia-responsive nanocarriers, may selectively dismantle this tripartite axis, offering a promising avenue to reverse the treatment-refractory phenotype of advanced HCC.

#### Infiltration of the marginal zone

At the invasive margin of HCC, NETs exhibit a spatial gradient distribution along the tumor-stroma interface (TSI) [177], forming a topological colocalization structure with α-SMA+/PDGFRβ+ activated HSCs and type I/III collagen fibers [178]. Concurrently, NET-derived CXCL1 recruits CD33+/Arg1+ myeloid-derived suppressor cells via the CXCR2-pSTAT3 signaling axis, establishing an immune-privileged niche characterized by high PD-L1 expression and suppressed CD8+ T-cell infiltration [179]. Collectively, these orchestrated interactions propel the malignant cascade of tumor invasion and transorgan metastasis. This TSI-focused NET-stroma-immune crosstalk highlights the TSI as a critical therapeutic target. Strategic co-targeting of NET-derived MMP9 (via selective inhibitors like rebimastat) and the CXCL1–CXCR2 axis (using reparixin) could disrupt both the structural integrity of the invasive front and the immune-suppressive barrier, potentially sensitizing aggressive HCC to anti-metastatic therapies and immunotherapies. Such approaches may prove pivotal in halting the spatial progression of HCC from localized invasion to systemic dissemination.

#### Perivascular region

Within the perivascular region of HCC, NETs specifically ensheath portal vein endothelial cells (PVECs) and liver sinusoidal endothelial cells (LSECs) [180], co-aggregating with activated platelets (CD61+/P-selectin+) [181] to form fibrin-platelet microthrombus complexes. NETs contribute to this pro-metastatic vascular niche through coordinated mechanisms: their associated VEGF-A/platelet factor 4 (PF4) complexes enhance vascular permeability [182], facilitating integrin αvβ3-mediated transendothelial migration of tumor cells [183]. Concurrently, NETs activate the thrombin-protease-activated receptor (PAR) signaling axis via tissue factor (TF)-FVIIa coagulation cascades [184], inducing upregulated expression of P-selectin glycoprotein ligand-1 (PSGL-1) on circulating tumor cells (CTCs). This upregulation accelerates fibrinogen-dependent anchoring of CTCs within liver sinusoids [185] and STAT3-driven metastatic niche formation [186]. Collectively, these interactions establish a vascular microenvironment primed for metastatic dissemination. The multifaceted role of NETs in vascular niche remodeling underscores the potential of targeting NET-vascular endothelial crosstalk. Combining anti-P-selectin antibodies (to disrupt platelet-NET adhesion) with PAR antagonists (to block coagulation-driven CTC anchoring) could dismantle this pro-metastatic axis, offering a novel strategy to intercept HCC hematogenous spread and limit distal organ colonization. Such approaches may prove particularly impactful in preventing early metastatic seeding, a critical unmet need in advanced HCC management.

#### Satellite nodule area

Within the satellite nodule region of HCC, NETs encase micrometastases in a “cloud-like” three-dimensional topological structure, exhibiting spatial co-localization with the pre-metastatic niche (PMN) marker S100A8/A9 heterodimer [187, 188]. Mechanistically, NETs release high-mobility group box 1 (HMGB1) to activate the TLR4/9-MyD88-NF-κB pathway in dormant tumor cells [189–191], inducing SOX2/OCT4 transcriptional reprogramming [192] and driving tumor cell re-proliferation. Concurrently, NETs secrete IL-8/IL-1β to form chemokine gradients [78], recruiting pro-angiogenic neutrophils via the CXCL8/IL-8 signaling axis [193] and upregulating CSF1-mediated M2 macrophage polarization [194]. These coordinated interactions generate a pro-metastatic cytokine storm, ultimately facilitating malignant remodeling of distal organ microenvironments and metastatic colonization. This intricate crosstalk between NETs and satellite nodules highlights the satellite niche as a critical therapeutic vulnerability. Targeting HMGB1–TLR4/9 signaling (e.g., via glycyrrhizin) alongside inhibition of the CXCL8/IL-8 axis (using reparixin) could disrupt both tumor cell reactivation and stromal recruitment, offering a dual-pronged strategy to suppress metastatic outgrowth. Such interventions may prove transformative in halting the progression of micro-metastases to clinically overt secondary tumors, a key challenge in HCC management.

### Multidimensional network collaboration shapes the spatial heterogeneity of NETs

#### Mechanical microenvironment driving

Within the mechanical microenvironment of HCC [195], gradients in matrix stiffness and fluid shear stress synergistically drive the spatiotemporally specific formation of NETs [196]. Portal vein shear stress triggers Ca^2 +^ influx in neutrophils through the mechanosensitive ion channel Piezo1 [197], activating the CaMKII-NFAT pathway [198] to induce upregulated PAD4 expression and citrullination. Concurrently, ROS bursts [199] further amplify NET release. This mechanical-biochemical signal coupling forms a positive feedback loop during tumor progression, driving HCC invasion and metastasis. Similar to the classification of GBM into immune hot and cold subtypes based on immune gene expression [123], HCC with high NET deposition in the invasive margin (characterized by elevated YAP/TAZ activity and ECM stiffness) can be classified as an immune “hot” subtype with increased effector memory CD8 T cell infiltration, while the tumor core with dense NETs exhibits an immunosuppressive “cold” phenotype [49, 123]. This subtype-specific difference further supports the spatiotemporal heterogeneity of NETs in regulating TME mechanics and immunity. The interdependence of mechanical cues and NET dynamics highlights the mechanical microenvironment as a pivotal therapeutic target. Such approaches may complement conventional therapies by addressing the physical determinants of HCC aggressiveness, offering a novel dimension to metastatic HCC management.

#### Metabolic reprogramming Regulation

In the context of metabolic reprogramming regulation in HCC, the hypoxia-lactate axis and lipid metabolic remodeling form a dual-driver network [200]. In the hypoxic tumor core, HIF-1α-induced neutrophil glycolysis produces lactate, which triggers NETosis via the HMGB1–TLR4-p38 MAPK axis [201, 202] and forms a metabolic-immune barrier by suppressing CD8+ T cells and upregulating PD-1/CTLA-4 [203–206]. At the invasive margin, enhanced FAO generates Ac-CoA, which induces H3K27ac modification via p300/CBP [207], activating neutrophil NF-κB/STAT3 to promote IL-6/IL-8 secretion and CXCR4-mediated pro-metastatic transformation [208, 209].

This spatial metabolic-epigenetic coupling fuels HCC progression and resistance. Spatially targeted interventions—combining hypoxia-activated HIF-1α inhibitors (core lactate suppression) with FAO antagonists (margin Ac-CoA blockade)—may reverse metabolic immune evasion, sensitize HCC to immunotherapy, and bridge metabolic targeting with precision immuno-oncology.

#### Immune editing network

Within the immune editing network of HCC, NETs mediate multi-dimensional immune evasion through regional immunosuppression and intercellular crosstalk. In the tumor core, NETs suppress CD8^+^ T cell proliferation and effector function via the PD-L1/PD-1 axis [210] and the indoleamine 2,3-dioxygenase (IDO)-kynurenine pathway [211]. Perivascular NETs, meanwhile, recruit Foxp3+ Tregs through C3a/C5a complement signaling [212], synergistically inhibiting the maturation of antigen-presenting cells (APCs) [213]. At the level of intercellular communication, NETs facilitate signaling between neutrophils and cancer cells via NE release, where the NE-PAR2 axis modulates miRNA expression (miR-34a/miR-146a) in cancer cells. This regulation impacts CD24 expression, enhancing macrophage phagocytosis, recruitment, and M1 polarization [214]. Additionally, NETs induce CAFs to secrete IL-6/IL-10 by inhibiting the PTEN/PI3K-Akt pathway [215], which in turn activates the STAT3/SOCS3 axis to promote IL-17A release [107, 216]; this further triggers NET formation, establishing a “NETs-CAFs-immunosuppression” vicious cycle that drives HCC immune editing and therapeutic resistance. This intricate network highlights NETs as central coordinators of immune evasion, necessitating combinatorial strategies that co-target NET-mediated immunosuppressive pathways. For instance, combining NET-dissolving agents (e.g., DNase I) with dual PD-1/IDO inhibitors could disrupt both T cell exhaustion and metabolic immune checkpoints, while targeting the NE-PAR2 axis might break the intercellular crosstalk sustaining the vicious cycle. Such approaches hold promise for reinvigorating anti-tumor immunity and overcoming resistance to current immunotherapies in advanced HCC.

#### Proteinase-cytokine network

At the invasive front of HCC, NETs precisely orchestrate malignant tumor behavior through a dynamic network of proteases and cytokines. In terms of protease-receptor interactions, NET-associated cathepsin G specifically cleaves the CXCL12 chemokine to generate a truncated ΔCXCL12 variant [217]. This fragment activates the PI3K/Akt/mTORC1 signaling axis in tumor cells via the CXCR4 receptor [218, 219], driving chemotactic directional migration. Collectively, this spatiotemporally coupled protease regulatory network serves as a molecular engine propelling HCC invasion and metastasis. Targeting this network’s key nodes, such as MMP9–TIMP1 equilibrium or cathepsin G-mediated CXCL12, processing could disrupt the spatiotemporal control of invasive behavior, offering a promising avenue to halt HCC metastatic progression.

### Specific regulation scheme based on spatial heterogeneity of the Nets

#### Regional targeted drug delivery system

To address the microenvironmental heterogeneity of HCC, regionally targeted drug delivery systems enable precise intervention through spatiotemporal-specific design. In the tumor core, hypoxia-responsive nanoparticles—such as nitroimidazole-modified PLGA liposomes [220], encapsulating DNase I and the PD-L1 inhibitor atezolizumab trigger drug release in hypoxic microenvironments, simultaneously degrading NETs and blocking the PD-L1/PD-1 immune checkpoint. At the invasive margin, MMP9 enzyme-sensitive prodrugs are engineered to leverage locally elevated MMP9 activity for site-specific cleavage and drug release [221], achieving spatial targeting of cytotoxic agents. This strategy, driven by microenvironment-responsive intelligent drug release mechanisms, transcends the spatiotemporal limitations of conventional therapies, offering a multidimensional precision treatment paradigm for HCC. Such microenvironment-adaptive systems not only enhance therapeutic efficacy by concentrating agents at pathological niches but also minimize off-target toxicity, laying the groundwork for combinatorial strategies that co-target diverse HCC subregions to overcome intratumoral resistance (Table 3).

**Table 3:** Comparison of the mechanism of action, clinical stage and limitations of existing NETs-targeting drugs

Targeted drug	Mechanism	Clinical stage	Limitation
BB-Cl-amidine	PAD4 inhibitor	Preclinical research and human trials have not yet been initiated: Lack of Phase I clinical data at this stage	Oral bioavailability is low. It non-specifically inhibits PAD2, inhibits the differentiation of myeloid progenitor cells and the function of T cells, and is difficult to penetrate the core area of the tumor.
DNase I	Specific cleavage of the phosphodiester bonds in the DNA backbone	The field of tumor metastasis inhibition is still in the pre-clinical stage.	The cutting efficiency of the highly condensed NETs-DNA/histone complex is low, and it is unable to inhibit the formation of new NETs.
Sivelestat	Neutrophil elastase inhibitor	Adjuvant Therapy for Pancreatic Cancer-Phase Ib Clinical Trial (NCT04825288) (Combining Gemcitabine to Reduce CA19-9 by ≥50%)	The first-pass effect is significant and requires continuous intravenous infusion. It has cross-inhibition effects on proteinase 3 (PR3). Tumor-associated macrophages (TAMs) compensate for the NE function through MMP-9.
Verteporfin	Hippo pathway antagonists competitively bind to the TEAD binding domain of YAP/TAZ, thereby disrupting the formation of the YAP-TEAD transcriptional complex.	Pancreatic Cancer Adjuvant Therapy I/II Phase Trial (NCT03033225)	The anti-tumor effect is relatively weak under non-light exposure conditions. The penetration depth of the tumor core is less than 2 mm. The plasma t₁/₂ after intravenous injection is approximately 5 hours, and the retention time in solid tumors is insufficient.
Cilengitide	αvβ3/αvβ5 integrin inhibitor	Phase III trial of glioblastoma (GBM) stage III failed (2013) (NCT00689221); Phase II trial of head and neck squamous cell carcinoma (HNSCC) completed (combination of cisplatin/chemotherapy resulted in a 3-year OS of 59%); Phase I trial of metastatic castration-resistant prostate cancer completed (NCT00073229) (tolerable dose 20 mg/kg, PSA response rate 18%).	Only blocking αvβ3/αvβ5 has no inhibitory effect on αvβ6/αvβ8, and the tumor distribution rate in the glioma model is less than 0.1%. The competitive antagonistic effect of vitronectin
NF-56-EJ40	Highly selective succinate receptor 1 (SUCNR1/GPR91) antagonist	Liver metastasis of colorectal cancer: In the Phase I trial (NCT05521858): Dose escalation completed (maximum tolerated dose 360 mg BID), preliminary tracer study showed liver-specific enrichment; Pancreatic ductal adenocarcinoma, preclinical proof-of-concept (combination with gemcitabine led to tumor regression rate of 74% in gemcitabine-resistant models); Multiple myeloma, mechanism study (inhibiting IL-6 secretion of bone marrow stromal cells, reversing bortezomib resistance)	Insufficient metabolic stability, species-specific effect (the inhibitory activity against SUCNR1 is 400 times weaker in dogs, hindering toxicological research); interference with renal clearance (the normal physiological function of SUCNR1 is involved in the regulation of the juxtaglomerular apparatus, and renin release is inhibited, leading to the risk of hyperkalemia)
Metformin	LDHA inhibitor	Adjuvant therapy for breast cancer Phase III (NCT01101438) (DFS of patients with diabetes was prolonged by 9.3 months); Phase II for colorectal cancer prevention completed (adenoma recurrence rate decreased by 23%, 40% reduction in the group using aspirin in combination); Negative results of Phase II for castration-resistant prostate cancer did not improve rPFS (combination of enzalutamide vs monotherapy, NCT02339168); Phase I/II for glioblastoma ongoing.	Dose-effect contradiction: Antitumor drug concentration needs to be ≥ 10 μM (the conventional hypoglycemic dose is only 1–2 μM); Metabolic heterogeneity influence (tumors with LKB1 gene deletion do not respond to AMPK activation, and the effective rate of pancreatic cancer model is <10%); Gastrointestinal side effects limitation (the incidence of grade 3 diarrhea in the high-dose group is 28% vs 4% in the placebo group); Lack of biomarkers (existing indicators such as p-AMPK have a weak correlation with clinical benefit).
NPS-2143	GPR81inhibitor	Termination of medullary thyroid cancer stage II; Breast cancer with bone metastasis, stage Ib/II (NCT04804761); Prostate cancer with castration resistance, preclinical proof-of-concept (inhibiting the proliferation of PSA-positive cell lines (IC₅₀ = 3.2 μM), increasing the sensitivity of enzalutamide by 4 times); Multiple myeloma bone disease, mechanism study (reversing the activation of osteoclasts induced by bortezomib).	Risk of blood-brain barrier penetration (activation of central CaSR leading to epileptic seizures); calcium metabolism disorder (dose-dependent hypercalcemia); off-target effect; drug efficacy heterogeneity.
β-Aminopropionitrile	LOXinhibitor	Pancreatic ductal adenocarcinoma stage I is ongoing (NCT04168937) (combined with albumin paclitaxel: initially achieving tumor stroma remodeling); intrahepatic cholangiocarcinoma, preclinical proof of concept; colorectal cancer liver metastasis, mechanism study (inhibiting LOXL2-mediated Snail1 stability); soft tissue sarcoma, preclinical efficacy assessment (improving doxorubicin penetration within tumors).	Acute toxicity risk (including copper amine oxidase MAO, causing cardiovascular toxicity); poor metabolic stability, requiring continuous infusion for maintenance; off-target effect (non-specific inhibition of copper-zinc superoxide dismutase); paradox of fibrosis reversal (excessive inhibition leads to disruption of vascular structural integrity).
Piezo1 monoclonal antibody	Targeting the mechanically sensitive ion channel Piezo1	Hepatocellular carcinoma Phase I trial (NCT05354258) (The first human trial showed a ≥ 20% reduction in portal vein pressure gradient); Pancreatic ductal adenocarcinoma, preclinical proof-of-concept (Combining gemcitabine reduced tumor hardness by 62%); Glioblastoma, mechanism study (Inhibiting Piezo1-TRPV4 synergistic activation delays temozolomide resistance); Breast cancer bone metastasis, preclinical efficacy assessment (Reducing mechanical activation of osteoclast precursor cells).	Poor tissue permeability (antibody molecular weight limits penetration into dense interstitium); compensatory pathway activation (upregulation of TRPC1/TRPC6 channel expression); species cross-reactivity limitation (weak binding force 10 times lower to crab-eating monkey Piezo1, hindering toxicological research); hemodynamic interference (blood pressure fluctuations, decreased systolic pressure).
GsMTx4	Broad-spectrum mechanically-sensitive ion channel inhibitor	Glioblastoma, preclinical proof-of-concept (combined with temozolomide to penetrate the blood-brain barrier); Pancreatic ductal adenocarcinoma, preclinical study (inhibiting the mechanical activation of CAFs, improving gemcitabine tumor penetration); Breast cancer lung metastasis, mechanism study (blocking the survival signals of circulating tumor cells to the mechanical compression of pulmonary capillaries); Osteosarcoma, preclinical efficacy assessment (inhibiting the activation of osteoclast Piezo1, reducing osteolytic lesions).	The polypeptide has poor stability and is easily degraded by proteases; the channel has non-selective inhibition (simultaneously blocking the effects on cardiovascular homeostasis of TRPC6); the tumor penetration is insufficient (with a limited distribution within solid tumors); compensatory signaling activation (mechanical force signals escape through the integrin β1-FAK axis).
GSK2837808A	Selective lactate dehydrogenase A (LDHA) allosteric inhibitor	Triple-negative breast cancer stage I/II (NCT04257335) (combined with atezolizumab: objective response rate ORR reached 28%); I stage dose escalation for non-small cell lung cancer (tolerance dose 800 mg BID, compliance rate of efficacy markers 89%); colorectal cancer with liver metastasis, preclinical proof-of-concept (inhibiting the activation of hepatic stellate cells and improving FOLFOX penetration); glioblastoma, mechanism study (reversing the O6-methylguanine repair escape induced by temozolomide)	Metabolic compensation escape (activating the AMPK-ULK1 autophagy pathway); Cardiac toxicity risk (inhibiting LDHA in cardiomyocytes leads to QTc prolongation); Bioavailability limitation (low oral absorption rate, affected by the intestinal ABCB1 efflux protein); Tumor heterogeneity response (low LDHA expression in tumors leads to poor response).
Etomoxir	Selective carnitine palmitoyltransferase 1 (CPT1) inhibitors, targeting the fatty acid oxidation (FAO) pathway	Phase I trial of triple-negative breast cancer (NCT04275435) (combined with paclitaxel: increased disease control rate, decreased plasma β-hydroxybutyric acid level); preclinical proof-of-concept for ovarian cancer (inhibiting tumor stem cells FAO, delaying platinum resistance); glioblastoma, mechanism study (destroying FAO in blood-brain barrier endothelial cells, improving temozolomide permeability); leukemia, preclinical drug efficacy assessment (targeting FAO-dependent of leukemia initiating cells).	Cardiac toxicity risk (inhibiting CPT1B in cardiomyocytes); Metabolic compensation failure (activating ACLY-mediated acetyl-CoA synthesis); Bioavailability limitation (significant first-pass effect of oral administration, requiring high-dose and frequent administration); Tumor heterogeneity response (overexpression of CPT1C subtype in tumors leads to drug resistance, such as in prostate cancer).

#### Mechanical signal Intervention

Interventions targeting the mechanical signaling network in HCC focus on dual targeting of matrix stiffness regulation and fluid shear stress blockade. For fluid shear stress blockade, anti-Piezo1 monoclonal antibodies [222] or GsMTx4 [223] suppress the Ca^2+^-ROS signaling axis and membrane potential changes activated by portal vein shear stress, reducing the incidence of NETosis and NET-driven circulating tumor cell (CTC) adhesion. This combinatorial strategy achieves mechanical reprogramming of the tumor microenvironment through “structural-mechanical” dual-dimensional intervention, providing a mechanistically defined therapeutic paradigm for inhibiting HCC invasion and metastasis. Such mechanical network disruption not only normalizes the physical properties of the HCC niche but also synergizes with immunotherapies by alleviating mechanical barriers to immune cell infiltration, highlighting its potential as a foundational strategy in multimodal HCC management. (Table 4).

**Table 4:** Nets target delivery system, mechanism of action, and objectives of action

Delivery system	Example	Mechanism	Purpose
Nanodelivery system	MMP2/9-sensitive micelles	Utilizing the highly active and specific cleavage of MMP9 in local areas to release drugs	Specific regional spatial targeted drug delivery
	Biomimetic nanocarriers of neutrophil membrane	Utilizing the CXCR1/2 chemokine receptors on the surface of neutrophils, actively target the regions where NETs are concentrated	Precise delivery of PAD4 inhibitors (such as BB-Cl-amidine) inhibits histone H3 citrullination and blocks NETosis.
Dynamic Monitoring Intelligent Delivery System	^6 8^ Ga-labeled anti-CitH3 monomer PET-MRI	By detecting the MPO-DNA complex and citrullinated histone H3 (CitH3) in the circulating fluid through liquid biopsy, and combining with PET-MRI imaging, the spatiotemporal heterogeneity of the spatial distribution of NETs can be precisely located in real time.	Dynamically analyze the spatial distribution characteristics of NETs, providing real-time data support for subsequent precise intervention and optimizing the treatment plan.
Microenvironment-responsive delivery system	Nitroimidazole-modified PLGA liposomes Liposomes encapsulating DNase I and the PD-L1 inhibitor atezolizumab	There is a hypoxic microenvironment in the tumor core region. Under hypoxic conditions, the nitroimidazole group triggers the disintegration of liposomes and releases the drugs.	Simultaneously degrade NETs and block the PD-L1/PD-1 immune checkpoint.
	pH/ROS dual-sensitive polymer carrier Nanocarrier carrying DNase I and Sivelestat	The acidic pH and high ROS levels in the NETs enrichment area trigger the disintegration of the carrier structure, releasing the drugs.	Local degradation of NETs scaffolds (DNase I) and inhibition of neutrophil elastase (Sivelestat) are employed to block the immune suppression and matrix degradation mediated by NETs, and this approach is applicable to inflammatory active areas.
Spatial transcriptome-guided biomimetic delivery	Based on the 10x Genomics Visium technology to locate the regions with high expression of NETs-related genes (such as MPO, PAD4), combined with the neutrophil membrane biomimetic nanocarriers	By using single-cell spatial transcriptomics to create a three-dimensional hotspot map of NETs, and by using biomimetic carriers (simulating the membrane of neutrophils) to target and deliver drugs to the CXCR1/2 positive regions.	By integrating spatial omics data, a “monitoring-imaging-intervention” closed loop is achieved, precisely inhibiting the generation of NETs and avoiding damage to normal tissues.
Mechanical signal-responsive delivery system	Matrix stiffness responsive nano-delivery carrier MMP2/9 sensitive micelles	In the highly rigid region of ECM, the activity of MMP2/9 enzymes increases, triggering the release of drugs (such as PAD4/NE inhibitors).	Disrupt the mechanical signal transduction axis (such as integrin αvβ3-FAK-YAP/TAZ), and reverse the fibrosis feedback loop mediated by NETs.
	Fluid shear force-blocking carrier Monoclonal antibody-conjugated nanoparticles targeting the mechanically sensitive ion channel Piezo1	The shear force of portal vein blood flow activates the Piezo1 channel in neutrophils. Anti-Piezo1 antibodies block the influx of Ca^2 +^ and the subsequent NETosis signaling pathway.	Inhibit the release of NETs by circulating neutrophils in the perivascular area, reduce the adhesion of CTCs and the formation of pre-transmission ecological niches.

#### Metabolic-immune synergistic targeting

In HCC, metabolic-immune synergistic targeting strategies integrate lactate metabolism intervention and lipid reprogramming blockade to form a cross-scale regulatory network. Within the tumor core, combined application of the LDHA inhibitor GSK2837808A [224] and anti-PD-1 antibodies (e.g., pembrolizumab) alleviates lactate-mediated inhibition of CD8+ T cell mitochondrial complex I by suppressing glycolytic end-product lactate accumulation. This dual approach concurrently reverses lactate-GPR81-driven NET formation and blocks the PD-1/PD-L1 immune checkpoint, dismantling the metabolic-immune barrier. At the invasive margin, inhibition of neutrophil fatty acid oxidation (FAO) via the CPT1a inhibitor etomoxir [225] reduces acetyl-CoA (Ac-CoA) levels, thereby suppressing H3K27ac epigenetic modification [207]; this reverses the neutrophil IL-8/CXCR1 pro-invasive phenotype [226, 227] and MMP9-mediated matrix degradation [228]. Through bidirectional “metabolic remodeling-immune activation” synergy, this strategy enables multi-dimensional regulation of the tumor microenvironment, offering an innovative metabolic intervention paradigm for HCC treatment. Such metabolic-immune crosstalk targeting not only enhances anti-tumor immunity by relieving metabolic constraints but also provides a framework to overcome therapy resistance driven by nutrient-deprived niches, underscoring its potential for clinical translation in advanced HCC.

#### Combined therapy guided by Spatialomics

To address the microenvironmental heterogeneity of HCC, single-cell spatial transcriptome navigation combined with biomimetic delivery systems forms a precision therapeutic closed loop. Leveraging 10x Genomics Visium spatial transcriptomics to map NET-associated gene clusters—such as regions with high expression of MPO, PAD4, and CXCL8 coupled with single-cell ATAC-seq analysis of chromatin accessibility [229], three-dimensional NET hotspot maps are constructed. These maps guide spatiotemporal dose optimization for stereotactic body radiation therapy (SBRT) or NIR-II photothermal therapy. Concurrently, neutrophil membrane biomimetic nanocarriers [230] are engineered, utilizing surface CXCR1/2 chemokine receptors for active targeting of NET-enriched regions to precisely deliver the PAD4 inhibitor BB-Cl-amidine, thereby blocking the NETosis vicious cycle via inhibition of histone H3 citrullination [143]. This strategy, empowered by bidirectional “spatial omics navigation-biomimetic carrier delivery,” transcends the blind-spot limitations of conventional therapies, offering a multimodal solution for NET-associated tumor intervention. Such integration of high-resolution spatial mapping and cell-mimetic targeting not only enhances therapeutic precision but also paves the way for adaptive interventions that dynamically respond to evolving NET landscapes, marking a critical advance in personalized oncology for HCC (Table 3).

## The spatiotemporal heterogeneity of NETs remodels the TME through dynamic energy landscapes, thereby driving the maintenance of tumor stem cell (CSC) properties

### The impact of the spatiotemporal heterogeneity of NETs on the energy landscape

NETs exhibit distinct distribution patterns across different regions of HCC and throughout disease progression [231]. Notably, NET density and structure differ significantly across HCC stages and etiologies. For instance, NETs are sparse and scattered in early HCC, but become dense, compact, and widely distributed in progressive and advanced HCC [232]. In addition, NET formation is more prominent in HBV-related HCC than in non-viral etiologies such as NASH-related HCC, likely due to stronger chronic inflammation and neutrophil activation in viral hepatitis [233]. Through mechanisms such as metabolite release, signaling pathway activation, and modulation of the mechanical microenvironment, they contribute to the formation of regionally specialized metabolic niches.

#### Time dimension

In the HCC microenvironment, NETs influence dynamic changes in the energy landscape across distinct disease stages. During early-stage HCC (metabolic homeostasis phase), neutrophils primarily rely on oxidative phosphorylation (OXPHOS) [234], with NETosis suppressed via the AMPK-mTORC1 pathway to maintain metabolic balance between glycolysis and OXPHOS, accompanied by physiological levels of ROS [235]. In progressive HCC (metabolic imbalance phase), tumor cells secrete G-CSF/CXCL1 to recruit neutrophils [236]. Lactate then activates PAD4 via the GPR81-p38 MAPK axis [237, 238], driving explosive NET release and inducing a Warburg effect-dominated metabolic imbalance [239, 240]. Concurrently, mitochondrial miR-181a-5p inhibits electron transport chain (ETC) function and activates glycolysis in HCC cells, further promoting tumor progression [241]. In advanced/therapy-resistant HCC (metabolic stress phase), chemotherapy-induced γH2AX+ DNA damage [242] and succinate accumulation activate NETs via the SUCNR1-PKCα pathway [243], leading to release of NE and cathepsin G (CatG), which degrade MCL-1 [244]. Simultaneously, mitochondrial membrane potential collapses [245], the succinate/α-ketoglutarate (α-KG) ratio becomes imbalanced, and the tricarboxylic acid (TCA) cycle is arrested [246], forcing tumor cells to depend on fatty acid oxidation (FAO) for energy [247] and establishing a therapy-resistant metabolic phenotype. This “metabolic plasticity-temporal heterogeneity” coupling network reveals the energy regulatory essence of NET-driven HCC progression, providing a theoretical basis for targeting metabolic vulnerabilities. Such stage-specific metabolic reprogramming by NETs underscores the need for time-sensitive therapeutic strategies that disrupt stage-specific energy pathways, offering a framework to intercept HCC progression at critical metabolic checkpoints.

#### Spatial dimension

In the HCC microenvironment, NETs drive spatial heterogeneity in the metabolic landscape through their distinct distribution patterns. Within the tumor core (hypoxic-acidic niche), hypoxia enhances neutrophil glycolysis via the HIF-1α/GLUT1 axis [248, 249]. NETs released here secrete lactate [191] and succinate [243], which inhibit mitochondrial complex I activity in CD8^+^ T cells, establishing an acidosis-dependent immunosuppressive barrier. Metabolic homeostasis is maintained via MCT1/MCT4 lactate transporters [250]. At the invasive margin (metabolic hybrid zone), NETs activate the NOX4-ROS pathway in CAFs through MMP9-mediated ECM degradation [251–253], promoting glutaminase (GLS1) activity and glutamine catabolism [254]. Concurrently, NET-DNA enhances glycolysis in CSCs via the TLR9–PI3K/Akt/mTOR axis [255], driving competitive glucose/glutamine utilization and a pro-invasive phenotype. In the perivascular region (high shear stress-oxidative stress niche), shear stress triggers NETosis via the Piezo1-Ca^2 +^ axis [197]. NETs encapsulate platelets, releasing PF4 to promote VEGF-A-mediated vascular leakage [256]. Simultaneously, elevated ROS levels induce mitochondrial permeability transition pore opening, forcing cells into glycolysis-dependent energy production [257]. This “spatial metabolic zonation-mechanical signaling coupling” network highlights how NETs orchestrate microenvironmental reprogramming critical for HCC progression. Targeting these niche-specific metabolic vulnerabilities—such as lactate shuttling in the core, glutamine addiction at the margin, or mechanosensitive pathways perivascularly, could provide a framework for spatially precise therapeutic interventions.

### Molecular mechanisms by which energy landscapes maintain tumor stemness

In the HCC microenvironment, NETs engage in a sophisticated interplay of metabolite release and epigenetic modulation to activate the self-renewal pathways of CSCs. This metabolite-epigenetic crosstalk mediated by NETs highlights a critical mechanism by which the HCC microenvironment sustains CSC populations. Targeting this complex interaction could potentially disrupt CSC-driven tumor progression and offer new therapeutic opportunities for HCC treatment.

#### Core region Lactic acid-HIF-2α Axis

Lactate released by NETs enters CSCs via the monocarboxylate transporter MCT1 [258], where it inhibits prolyl hydroxylase (PHD) to stabilize HIF-2α, thereby upregulating the stemness transcription factors Oct4 and Nanog [259]. Concurrently, lactate activates the GPR81-adenylate cyclase (AC)-PKA pathway [260], inducing phosphorylation of β-catenin at Ser552 to promote its nuclear translocation [261, 262] and enhance Wnt/β-catenin signaling [263]. This dual mechanism—linking metabolic cues to transcriptional activation of stemness programs—positions NET-derived lactate as a critical driver of CSC maintenance. A pan-cancer study identified TRAF5 as a key prognostic gene whose mutation correlates with shortened patient survival, and its expression is associated with stemness-related gene upregulation [49], which parallels the NETs-lactate-HIF-2α-Oct4/Nanog axis in HCC. Additionally, the identification of immune hub genes (e.g., CD54) in GBM [123] provides a reference for exploring NETs-associated stemness biomarkers in HCC. Targeting MCT1-mediated lactate uptake or GPR81-PKA signaling could disrupt this metabolic-epigenetic axis, offering a strategy to deplete the CSC pool and reduce HCC recurrence.

#### Lactic acid and Succinic acid-epigenetic Reprogramming

In regions of HCC enriched with NETs, low FOXP2 expression prevents KDM5A-mediated demethylation of H3K4me3 at the FBP1 promoter, thereby transcriptionally upregulating FBP1 [264]. This increase in FBP1 activity accelerates glycolysis, driving lactate accumulation and reinforcing the Warburg effect. Concurrently, succinate released in these niches activates the SUCNR1–STAT3 axis to induce DNMT1c [265], which catalyzes hypermethylation at the C/EBPα gene promoter [266], silencing the expression of differentiation-associated genes. This interplay of transcriptional and epigenetic regulation—coordinated by NET-associated metabolites—highlights how NETs sculpt a stemness-promoting metabolic-epigenetic landscape. Targeting these interconnected pathways, such as inhibiting FBP1 to reverse glycolytic dependency or blocking DNMT1c-mediated silencing, could disrupt the pro-tumorigenic niche sustaining HCC progression.

#### Synergistic regulation of mechanical Signals

NETs release NE and MMP9, which degrade basement membrane components [267], induce enhanced collagen fiber cross-linking, and increase extracellular matrix (ECM) stiffness. These mechanical property changes activate the YAP/TAZ transcriptional co-activators via the integrin β1-FAK-ROCK signaling axis [268], promoting the expression of stemness genes such as Sox2 and OCT4 [269]. Concurrently, the three-dimensional DNA meshwork formed by NETs induces morphological polarity changes in tumor cells through spatial constraint forces, activating the Piezo1 mechanosensitive ion channel. This triggers calcium oscillations and drives HIF-1α-mediated glycolytic metabolic reprogramming [270].

Taken together, this “metabolic-epigenetic-mechanical” multi-dimensional regulatory hub reveals the molecular mechanisms by which NETs drive the maintenance of CSC stemness, providing precise intervention targets for CSC-directed therapies. Disrupting this integrated regulatory network—for example, by combining MMP9/NE inhibitors with Piezo1 antagonists—could synergistically abrogate CSC stemness, offering a promising strategy to overcome treatment resistance in HCC.

## NETs-energy landscape axis-mediated microenvironment remodeling

### Immune metabolic inhibition

In the HCC microenvironment, NETs play a pivotal role in reshaping an immunosuppressive milieu through a sophisticated coupling of metabolite release and acid signaling.

#### T cell exhaustion

Lactate released by NETs inhibits IFN-γ secretion in CD8+ T cells via the GPR132-Myc pathway, promoting T cell exhaustion through the lactate-GPR132/Myc axis. Concurrently, it activates the aryl hydrocarbon receptor (AhR), inducing co-expression of PD-1 and TIM-3. NET-associated indoleamine 2,3-dioxygenase (IDO) metabolite kynurenine enhances AhR stability via deubiquitination, driving CD8+ T cell exhaustion through the kynurenine-AhR signaling pathway [271]. Additionally, NET-related succinate promotes Treg expansion via tricarboxylic acid (TCA) cycle metabolism and SUCNR1 signaling, with succinate accumulation suppressing IFN-γ and TNF-α expression in effector T cells [272]. This multi-pronged metabolic-immune suppression network underscores NETs as central coordinators of T cell dysfunction. Targeting these pathways, such as blocking GPR132-mediated signaling or inhibiting IDO/kynurenine metabolism, which could reinvigorate anti-tumor immunity, offering synergistic potential with checkpoint inhibitors in HCC.

#### Macrophage polarization

In the tumor microenvironment, circulating mitochondrial DNA (mtDNA) activates NETs via cGAS [273] and induces polarization of CD163+ M2-type tumor-associated macrophages (TAMs) through the TLR9-NF-κB/STAT3 pathway [274–276]. These TAMs secrete arginase 1 (Arg1) to deplete microenvironmental arginine [277], suppressing CD8+ T cell proliferation [278], while simultaneously releasing IL-6/VEGF-A to promote angiogenesis [279] and fibrosis. Taken together, NETs drive T cell exhaustion through multiple signaling pathways and synergistically construct an immune-privileged microenvironment via the “lactate-kynurenine-succinate” triple metabolic inhibitory axis coupled with TLR9-mediated M2 polarization. This forms a metabolic-immune co-inhibitory network that fuels HCC immune evasion and therapy resistance. Targeting this integrated network, for example, by combining mtDNA/cGAS-NET inhibitors with strategies to reverse M2 TAM polarization, which could dismantle immune privilege, offering a novel approach to enhance immunotherapy efficacy in advanced HCC.

### Matrix malignant transformation

In HCC progression, NETs drive matrix malignant transformation and pre-metastatic niche (PMN) formation via a “metabolism-signaling axis”.

#### CAF activation and positive feedback loops

NET-released TGF-β induces phosphorylation of SMAD2/3 to promote cancer-associated fibroblast (CAF) expression of α-SMA and fibronectin (FN1) [280, 281], activating the TGF-β/SMAD pathway and enhancing collagen deposition. NET-associated lactate activates IL-1β via the NLRP3-ASC-caspase1 axis, driving CAFs to secrete IL-6/VEGF-A and adopt a pro-fibrotic phenotype. Activated CAFs further secrete CXCL12, which promotes neutrophil infiltration and NET formation via the CXCR4–PI3K/Akt pathway [281], establishing a CXCL12–CXCR4 positive feedback loop and forming a “NETs-CAFs-ECM remodeling” vicious cycle.

#### Pre-metastatic niche (PMN) construction

Circulating NETs carry S100A8/A9, which upregulates VCAM-1 in endothelial cells of distant organs via the TLR4-MyD88-NF-κB pathway [282, 283]; this S100A8/A9–TLR4 axis recruits CD11b+Gr1+ bone marrow-derived cells (BMDCs) [284]. BMDCs secrete MMP2/9 to degrade the basement membrane [284] and release LOXL2 to catalyze collagen cross-linking [285], forming a fibrotic “nidus” that facilitates circulating tumor cell (CTC) colonization.

#### Mechanical-metabolic synergistic regulation

ECM stiffening in PMNs activates the integrin β1-FAK-YAP pathway via YAP/TAZ mechanotransduction, upregulating SOX2/OCT4 in CTCs to enhance stemness and drug resistance [286]. NET-released succinate upregulates mitochondrial biogenesis in CTCs via the SUCNR1–PGC1α axis, reprogramming the succinate-TCA cycle to promote metastatic lesion growth.

This “primary tumor-distant organ” cross-scale regulatory network reveals the pathological basis by which NETs drive systemic HCC progression. Targeting critical nodes—such as disrupting the NETs-CAFs feedback loop or blocking S100A8/A9-mediated PMN initiation could intercept metastatic cascades at multiple stages, offering a mechanistic framework for anti-metastatic therapies.

## Summary and therapeutic insights

The spatiotemporal heterogeneity of NETs constructs a dynamic energy landscape in HCC through metabolite gradients (lactate, succinate, kynurenine), mechanical signals (stiffness, shear stress), and immune regulatory networks, driving CSC stemness maintenance and malignant remodeling of the TME.

### “Metabolic-mechanical-immune” three-axis collaborative intervention

From a metabolic intervention perspective, combining the LDHA inhibitor GSK2837808A [287] with the succinate receptor antagonist NF-56-EJ40 [161] blocks the lactate-HIF-2α and succinate-SUCNR1 axes, inhibiting the glycolysis-oxidative phosphorylation metabolic coupling in CSCs. For mechanical regulation, application of the LOX inhibitor β-aminopropionitrile [288] disrupts stiffness-dependent stemness signaling. To restore immune function, hyaluronic acid-targeted nanoparticle-encapsulated DNase I [289] efficiently degrades NET-DNA, while combination with anti-PD-1 antibodies reverses T cell exhaustion and reshapes immune surveillance capacity. This multi-modal strategy, integrating metabolic checkpoint inhibition, mechanical normalization, and immune reactivation, addresses the spatiotemporal complexity of NET-driven HCC progression. Such combinatorial approaches hold promise for overcoming the adaptive resistance often observed with single-agent therapies, offering a synergistic framework to disrupt the “metabolism-mechanics-immunity” pathogenic network.

### Spatiotemporal synergistic effect

In the tumor core, metabolic interventions prioritize suppression of hypoxia-driven lactate accumulation and the Warburg effect, thereby reducing the concentration of immunosuppressive metabolites [224]. At the invasive margin, disruption of mechanical signaling pathways inhibits the EMT process. This region-specific therapeutic stratification aligns with the spatiotemporal heterogeneity of the HCC microenvironment, ensuring interventions target niche-specific vulnerabilities. By decoupling metabolic-immune crosstalk in the core while halting invasive reprogramming at the margin, such strategies could synergistically constrain both local tumor expansion and metastatic dissemination.

### Whole-body system

Within the systemic circulatory system, engineered immune nanomedicines [290] can be deployed to achieve spatiotemporally specific clearance of circulating NETs, further blocking pre-metastatic niche formation.

Taken together, this strategy offers a novel paradigm for overcoming heterogeneous drug resistance in HCC through a spatiotemporally targeted intervention network. Future efforts should focus on translating these insights into patient-specific therapeutic regimens tailored to molecular subtypes, potentially integrating multi-omics profiling to refine stratification and optimize response rates across diverse HCC populations. Such personalized approaches could maximize the clinical impact of NET-targeted therapies by accounting for inter-individual variations in TME dynamics and metastatic potential.

### Critical analysis of the dual role of NETs in HCC

Most preclinical and clinical studies in HCC support the pro-tumorigenic paradigm of NETs: for instance, NET-derived DNA-LL37 complexes activate the TLR9–PI3K/Akt pathway to induce EMT and CSC expansion [135], while NET-associated NE degrades TKIs to promote drug resistance [176], and such pro-tumor effects dominate in chronic inflammatory microenvironments where sustained NET release induces immune exhaustion and ECM stiffness [291]. In contrast, anti-tumor effects of NETs are more prominent in acute inflammatory settings, where transient NET formation triggers rapid immune cell recruitment before adaptive immunosuppression is established [59, 292]. This functional duality is linked to distinct NET characteristics and formation pathways: pro-tumor NETs are enriched in proteases (NE, MMP9) [293, 294]and immunosuppressive cytokines (IL-10, ARG1) [295], arising primarily from NOX2-dependent lytic NETosis (predominant in chronic inflammation) [296], while anti-tumor NETs preferentially contain antimicrobial peptides (LL-37) and pro-inflammatory cytokines (IL-12, CXCL10) that enhance anti-tumor immunity, generated via non-lytic NETosis induced by acute immune stimulation [297]. Additionally, the role of NETs varies with disease stage and HCC etiology: in early-stage HCC, NETs may limit tumor growth by trapping CTCs in liver sinusoids, but this protective effect is lost in advanced stages as NETs shift to promote pre-metastatic niche formation [298]; NETs derived from HBV-related HCC exhibit stronger pro-tumor effects via TLR9 activation, compared to those from NAFLD-related HCC (metabolic-induced NETosis), which show weak anti-tumor potential [114]. In summary, the dual role of NETs in HCC is not an inherent contradiction but a context-dependent phenomenon shaped by TME inflammation, NET component heterogeneity, disease stage, and etiological background. This highlights the need for precision targeting strategies that inhibit pro-tumor NETs while preserving potential anti-tumor effects, rather than global NET ablation, and future research should prioritize defining NET subtypes and their functional contexts to reconcile discrepancies and maximize the clinical benefit of NET-targeted therapies.

## Challenges and prospects

HCC therapy remains confronted with substantial challenges, primarily stemming from the complexity of tumor progression mechanisms and, notably, difficulties in spatiotemporal dynamic tracking. The spatiotemporal heterogeneity of NETs within the HCC microenvironment involves multi-dimensional crosstalk (e.g., mechanical, metabolic, and immune interactions), whose dynamic evolutionary processes cannot be fully captured by traditional static models such as 2D cell cultures. Furthermore, understanding of causal relationships remains elusive: the interplay between the energy landscape and NETs involves bidirectional regulation (e.g., lactate drives NETosis, while NETs release lactate), necessitating conditional gene knockout or optogenetic tools to clarify causal chains. Technical limitations persist, including insufficient spatial resolution—existing techniques like immunofluorescence and flow cytometry struggle to resolve the distribution and functional specialization of NETs at the micrometer scale, highlighting an urgent need for breakthroughs in spatial multi-omics (e.g., Visium [299], MERFISH [300]) and in vivo imaging (e.g., two-photon microscopy). Clinical sample heterogeneity is another hurdle: variations in genetic background, etiologies (e.g., hepatitis B, alcoholism), and treatment histories among HCC patients lead to highly variable NET characteristics, underscoring the need for large-scale single-cell spatiotemporal databases to standardize analyses. Clinical translation also faces multiple bottlenecks, such as off-target effects of targeting strategies—NET clearance (e.g., via DNase I) may disrupt physiological immune defenses [144], necessitating the development of spatiotemporally specific intervention tools (e.g., tumor microenvironment-responsive prodrugs). Regarding treatment timing and drug resistance, the dynamic adaptive properties of NETs may enable “therapeutic window escape,” requiring real-time monitoring of NET activity via liquid biopsies (e.g., circulating CitH3-DNA complexes).

Despite the promising synergistic potential of NET inhibitors with ICIs or anti-angiogenic drugs, several critical challenges hinder their clinical translation. Notably, this review predominantly focuses on the therapeutic potential of NETs as actionable treatment targets for HCC intervention, whereas the biomarker value of NETs in clinical practice is relatively underdiscussed. Specifically, we insufficiently elaborate the application of NETs in patient stratification and precise therapeutic decision-making, as well as their reference value for distinguishing patient subgroups and guiding individualized combination treatment allocation. First, single-agent ICIs already face low efficacy in HCC (ORR < 20%) due to NET-mediated immunosuppressive barriers in the TME-including physical obstruction of CD8+ T cell infiltration by NET-DNA scaffolds and T cell exhaustion induced by NET-associated PD-L1-yet validating the NET clearance-immune reactivation mechanism in clinical settings remains unmet, as preclinical findings lack direct clinical evidence across HCC etiological subtypes [301, 302]. Second, patient stratification for combination therapy is imprecise: while high circulating NETs levels correlate with poor ICI response, a standardized NETs activity scoring system is absent, leading to difficulties in identifying patients most likely to benefit from combined regimens [303]. Third, optimal administration regimens remain undefined: ICIs trigger explosive NET release within 72 hours post-treatment, which may counteract immune efficacy [304], but sequential dosing strategies have not been tested in HCC to determine safe and effective timing or dose ranges. Fourth, current animal models frequently fail to fully recapitulate the complex spatiotemporal and etiological heterogeneity observed in human HCC. Commonly used preclinical models, including orthotopic xenografts and genetically engineered mouse models (GEMMs), are mostly established under standardized genetic backgrounds and controlled environmental conditions. Although these models are valuable for exploring core molecular mechanisms, they cannot fully mimic the diverse etiological backgrounds of human HCC, such as chronic HBV/HCV infection, non-alcoholic steatohepatitis (NASH), alcohol-related liver damage, and metabolic disorders. Moreover, these models rarely reproduce the dynamic spatial distribution and stage-dependent structural changes of NETs that characterize human HCC progression [305, 306]. Consequently, the translational value of preclinical findings regarding NET-targeted therapies remains limited, and further optimization of clinically relevant animal models that integrate etiological diversity and tumor heterogeneity is urgently needed to bridge the gap between laboratory research and clinical practice. Moreover, issues such as insufficient funds and manpower, inadequate infrastructure, operational obstacles, and poor research management, lack of cooperation, lack of reproducibility of results, and lengthy ethical and regulatory approval processes will all act as obstacles for transferring animal models to human trials [307].

Future research should prioritize technological innovation to drive mechanistic insights, such as four-dimensional (4D) integrative analyses combining single-cell spatiotemporal transcriptomics, metabolomics (spatial metabolic imaging mass spectrometry), and mechanical profiling (atomic force microscopy) to construct a “time-space-function” multi-dimensional regulatory network of NETs. Additionally, organoid-NET co-culture systems—utilizing HCC patient-derived organoids (PDOs) co-cultured with neutrophils—could simulate dynamic interactions between NETs and tumor core, margin, or vascular regions, revealing region-specific regulatory mechanisms. Precision intervention strategies should be developed, including intelligent nanodelivery systems [308]—designing mechanically responsive (e.g., matrix stiffness-activated) or metabolically responsive (e.g., lactate/pH-sensitive) nanocarriers to achieve spatiotemporally precise drug release in NET-enriched regions—and synthetic biology tools, such as gene circuit-controlled neutrophils (e.g., light-regulated PAD4 expression), to selectively eliminate pathological NETs while preserving physiological functions. Further interdisciplinary integration and clinical translation are critical: strengthening computational models to predict therapeutic efficacy, such as using artificial intelligence (e.g., graph neural networks) to integrate multi-omics data, forecast resistance risks to NET-targeted therapies, and optimize combination regimens (e.g., NET inhibitors + immune checkpoint blockade); importantly, future studies should also attach greater importance to exploring NETs as novel diagnostic and prognostic biomarkers, developing unified NET evaluation criteria, and promoting the clinical application of NET-based stratification strategies to screen eligible patients and realize tailored therapy. It is also of great significance to enhance the connection between preclinical research and clinical research, such as microfluidic chips simulating metastatic niches to reconstruct the mechanical-metabolic microenvironment of HCC metastatic organs (e.g., lung, bone) and evaluate the role of NETs in cross-organ metastasis and intervention efficacy; validate NET inhibitor-ICI combination mechanisms via clinical trials: Design phase I/II trials enrolling diverse HCC subtypes to test DNase I + PD-1 inhibitors, with primary endpoints of ORR and DCR, and secondary endpoints including changes in NET markers and CD8+ T cell infiltration density; establish precision patient stratification and optimized dosing: Develop and prospectively validate a NETs activity scoring system to enrich phase II trials for advanced HCC patients with high NETs activity and PD-L1 positivity, improving treatment precision. Additionally, test sequential regimens and dose-escalation designs to define the optimal administration window for HCC, mitigating ICI-induced NET bursts and designing prospective clinical trials, such as phase II trials of NET-targeted therapies (e.g., DNase I + atezolizumab), combined with multi-parametric PET-MRI (e.g., ^68^Ga-CXCR4 probes) to dynamically assess correlations between NET clearance and tumor regression.

In summary, research on the spatiotemporal heterogeneity of NETs in HCC is at a critical transition from “phenomenological description” to “mechanistic intervention.” The YAP/TAZ/Hippo mechanotransduction pathway acts as the primary upstream driver, coordinating mechanical cues, metabolic reprogramming, and EMT; the lactate/HIF-2α axis (maintaining CSC stemness) and MMP9/TGF-β axis (driving matrix remodeling and invasion) are key downstream effectors, tightly linked to NET spatiotemporal heterogeneity; and PAD4 (NET formation), MMP9 (matrix degradation), Piezo1 (mechanosignal transduction), and MCT1 (lactate uptake)-all supported by preclinical/early clinical evidence-are the most druggable targets. Overcoming current challenges will depend on technological innovation (e.g., spatial omics, synthetic biology), interdisciplinary collaboration (computational biology, nanomedicine), and transformed clinical translation paradigms. With the widespread adoption of multi-dimensional analytical technologies (e.g., single-cell, spatiotemporal, mechanical, metabolic, and immune profiling) and breakthroughs in intelligent drug delivery systems, precision NET-targeted strategies hold promise as a new pillar for improving HCC prognosis, offering a novel paradigm to address the challenge of tumor heterogeneity.

## Data Availability

Data sharing is not applicable to this article as no datasets were generated or analysed during the current study.
